# Early detection of MDR *Mycobacterium tuberculosis* mutations in Pakistan

**DOI:** 10.1038/s41598-021-96116-x

**Published:** 2021-08-18

**Authors:** Ayma Aftab, Samia Afzal, Zahida Qamar, Muhammad Idrees

**Affiliations:** Division of Molecular Virology and Infectious Diseases, Centre of Excellence in Molecular Biology (CEMB), University of the Punjab, 87-West Canal Bank Road, Thokar Niaz Baig, Lahore, Pakistan

**Keywords:** Biotechnology, Microbiology

## Abstract

The result of improper treatment has led to the rise of Multidrug-resistant (MDR) strains. This concern still exists in Pakistan. In order to save energy, time and resources an early detection of resistant cases is imperative. Thus, a treated group of 100 isolates and a control group of 56 untreated isolates were studied. PCR and gene sequencing showed mutations at codon 531 and 513 in the *rpoB* gene. 12% of cases showed a double mutation in the *rpoB* gene. *katG* gene showed mutations at codon 315 and 299. 28.6% of the control group cases were positive for MDR whereas 100% of the treated group were positive for MDR. This study explores the significantly increasing ratio of MDR-TB among Pakistani population. This study provides prevalent MDR mutations among Pakistanis and suggests developing such molecular assays that are time and cost effective. **Importance:** Pakistan is a developing country and has fourth highest incidence rate of MDR-TB. The treatment of MDR-TB is the use of second line drugs that has severe side effects as well as it requires long time span. One of the strategies to control the spread of MDR-TB is to decipher the aberrations at molecular level in order to formulate potent drugs that can treat the patients within short span of time. Determining the mutation profile of MDR in Pakistani populations will open new horizons for the improvement of drug treatment regimens to make it more effective or for the development of novel potent drugs and vaccines to better treat the drug-resistant TB. Moreover, this study will be help in disease control program.

## Introduction

Pakistan is a developing country with a crowded population of 210 million people, out of which 1.5 million suffer from TB. Pakistan ranks sixth highest in the world for estimated number of tuberculosis cases. With the passage of time, the emergence of strains with drug-resistance and multiple drug resistance (MDR) has lethaly increased. MDR has become the leading health concern in Pakistan which currently ranks fourth among 27 countries highly burdened by MDR-TB^1^. According to a WHO report, 18% of MDR cases reported in the year 2018 were previously on treatment and 3.5% of cases did not receive prior treatment^2^. MDR is associated with the combined resistance to first line drugs, Rifampicin and Isoniazid. Rifampicin resistance (RIF^r^) is of particular epidemiological importance. Isoniazid resistance coexists with RIF^r^ in more than 90% of isolates^3,4^. Mutations in *rpoB* that confer resistance to Rifampicin hinder the formation of mRNA transcripts and RNA elongation, which thereby prevent binding to the beta subunit of RNA Polymerase^5,6^. Published data related to Rifampicin resistance illustrates 87 unique point mutations or short indels positioned in an 81-bp rifampin resistance-determining region (RRDR) that encodes 27 amino acids (codons 507 to 533). The *katG* gene encodes Catalase Peroxidase enzymes (~ 740-bp) that are indispensable for the conversion of Isoniazid, the first line drug, into its active form^7,8^ in *Mycobacterium tuberculosis.* Several genetic alterations in *katG* correspond to codons 315, 316, and 309^9^. A study based on a cohort of 163 patients in Belarus identified four types of mutations in codon 315: AGC→ACC (present in 85% of patients), AGC→AGG (2.3%), AGC→ACC (4.7%), and AGC→GGC (2.3%). One type of mutation was identified at codon 316 which was GGC→AGC (41.4%). Four types of mutations were found in codon 309: GGT→GGT (16.1%), GGT→GCT (9.2%), GGT→GTC (6.9%), GGT→GGG (2.7%)^9^. Moreover, a mutation at codon 315 was observed in 64% of cases around the globe^10^. Although isoniazid resistance due to *inhA* mutation has been observed but this resistance is of low level^11^. Various molecular techniques depend upon these genetic factors to diagnose MDR. Identification of MDR by detection of known mutations in *rpoB* and *katG* is as efficient as drug susceptibility testing (DST)^11^. MDR and extensive drug-resistant TB is not just limited to a few countries. It is now a world-wide phenomenon^12^. The most effective patient treatment and efficient disease control rely absolutely on the early detection of the drug-resistant *M.tuberculosis* strains.

Pakistan is the fourth highly vulnerable country in the world for MDR-TB^1^. It is, therefore, imperative that Pakistan should adopt such strategies as to control the disease at an early stage.

The type and severity of drug resistance in different populations lead to varied mutations. Therefore, extensive research deciphering changes at molecular level of mycobacterial genes will form the foundation for new treatment regimens. To identify mutations, automated DNA sequencing was adopted as it is a universally practiced method with high sensitivity and selectivity to characterize mutations^13–15^. This study was conducted to investigate the type and rate of occurrence of drug resistance found among *M. tuberculosis* clinical isolates that demonstrated an MDR phenotype. The objective of this study in Pakistan is to develop low-cost molecular assays depending upon already know mutations. This molecular test is time efficient and as accurate as drug susceptibility test (DST).

Our approach was to pick and select number of mutation sites that could be used for swift screening of isolates to detect MDR-TB in Pakistan.

## Results

### Amplification and nucleotide analysis

*rpoB* and *katG* genes’ selected regions were amplified by using the designated primers and were sequenced to unravel the mutations. The sequences were analyzed for aberrations against the reference sequence, GI: 57,116,681. The strain sequences were submitted to GenBank and accession numbers of the sequences are: JN315348.1, JQ394887-JQ394890, JN626460, JN626461, JN626462, JN626463, JN626464, JN626465, JN315348, JN315349, JN315350, JN315351, JN315352, JN315353, JN315354, JN315355, JN315356, and JN626466.

### Analysis of *rpoB* gene mutations

DNA sequence analysis of 156 M*. tuberculosis* samples taken from the people coming from different regions of Pakistan showed MDR*.* No polymorphism was observed in the control strain (H37Rv strain). 67% of the treated group had a mutation at codon 531 in *rpoB*. 21% of the treated group had an insertional mutation at codon 513 in *rpoB*. However, 12% of the treated group had mutation at both codons 531 and 513. Most of the mutations in MDR-TB strains were single nucleotide mutations in *rpoB*. 28.6% of the control group showed mutation at 531 in *rpoB*. No mutation was observed in the region outside the hot spot of *rpoB* gene (350 bp upstream of codons 507 to 533). The total number of cases found resistant to Rifampicin are provided in (Table 1). Table 2 shows the nucleotide and amino acid changes in the specific regions of *rpoB.* Table 3 compares the nucleotide sequence of *rpoB* gene with the control strain.

**Table 1:** Total number of multiple drug resistant cases in treated and control groups.

Cases	Category A *rpoB* [S531L] *katG* [S315T]	Category B *rpoB* [513Ins(Arg)] + *katG* [S315T]	Category C Double mutation *rpoB* [513Ins(Arg), S531L] + *katG* [S315T, G299S]
Treated (n = 100)	67 (67%)	21 (21%)	12 (12%)
Control (n = 56)	16 (28.6%)	Nil	Nil

**Table 2:** DNA sequencing data for *rpoB* mutations in MDR *M. tuberculosis* strains from Pakistan.

Altered codon in *rpoB* and *katG*	Nucleotide Change	Amino acid change
*rpoB* 531	TCG→TTG	Ser→Leu
*rpoB* 513	Ins (CGC)	Ins (Arg)
*katG* 299	GGC→AGC	Gly→Ser
*katG* 315	AGC→ACC	Ser→Thr

**Table 3:** Sequence comparison of *rpo* B gene (Mutation in hot spot).

H37Rv	520 CCG CTG TCG GGG TTG ACC CAC AAG CGC CGA CTG TCG GCG CTG
Mutated sequence	520 CCG CTG TCG GGG TTG ACC CAC AAG CGC CGA CTG TTG GCG CTG
	(Ser/Leu)
H37Rv	507 GGC ACC AGC CAG CTG AGC CAA TTC ATG GAC CAG AAC AAC CCG CTG
Novel Mutation	507 GGC ACC AGC CAG CTG AGC CGC CAA TTC ATG GAC CAG AAC AAC CCG CTG
	(Arg)
H37Rv	521 TCG GGG TTG ACC CAC AAG CGC CGA CTG TCG GCG CTG
Novel Mutation	521 TCG GGG TTG ACC CAC AAG CGC CGA CTG TTG GCG CTG
	(Ser/Leu)

### Analysis of *katG* gene mutations

MDR strains were analyzed via DNA sequencing of a 1120-bp *katG* fragment. In the *katG* gene, a mutation at codon 315 was found in 88% of treated cases where the wild type codon AGC was mutated to ACC (Ser→Thr). 12% of the treated group demonstrated double mutations, S315T and G299S. The latter mutation was found to be a novel mutation based on publicly available data. 28.6% of the control group showed S315T mutation. The H37Rv strain was used as a control strain and no mutations were found. Single nucleotide mutation was observed in the isolates. Table 1 shows the number of Isoniazid resistant cases. *katG* amino acid changes are shown in the Table 2.

### Statistical analysis

Depending upon the identification of different mutations in *rpoB* and *katG* during MDR-TB, the treated and the control groups were divided into 3 categories. Category A has individuals with mutations at *rpoB* [S531L] and *katG* [S315T], category B has individuals with mutations at *rpoB* [513Ins (Arg)], *katG* [S315T] and category C has individuals with double mutations at *rpoB* [513Ins (Arg), S531L], *katG* [S315T, G299S]. Mann Whitney U test was applied to this data to compare the two groups. The p-value was 0.03 that is significant.

## Discussion

Pakistan is one such country as lacks latest information and a vast majority of the masses lives under or close to the poverty line. These factors still exist even in the twenty-first century. This makes it easier to understand why mutated strains spring up. The standard procedure of observing MDR-TB is by finding resistance in *rpoB* and *katG* against rifampicin and isoniazid. Studies revealed that *katG* and *rpoB* are the commonly known genes that show mutations due to inconsistent medical treatment. For the study 156 samples were collected. 100 were of the patients already treated inconsistently and 56 of the patients who had not gone through any treatment. All the patients went through the standard operational procedure before the initiation of the treatment and conclusion.

Current research detected MDR-TB strains by culturing smear positive respiratory samples followed by drug sensitivity tests. It has now been established that mutations in *rpoB* gene and *katG* gene of *M. tuberculosis* are associated with resistance to Rifampicin and Isoniazid respectively. These mutations, along with novel ones, can be determined very easily and in a timely manner through sequence analysis. Previous studies from other countries affirm our findings in a Pakistani cohort that 95% of mutations appear in the 81-bp RRDR of *rpoB*. The most frequent mutations in RRDR are at codons 531, 526, and 516 which are listed in descending order of their occurrence in the population^6,16–18^.

Present study data is in accordance with the data presented by Thailand that the mutation in *rpoB*531 (58%) and *katG* Ser315 (100% of 38 strains) was commonly found in the study group^19^. An Australian study reported *rpoB* S531L (40.6%) and *katG* Ser315 (84.1%) as the most frequent mutations^17^ that has also been reported by a global survey conducted in 2015^18^. An Iranian study revealed the *rpoB* mutation at codon 531 (55.68%) and was among highly prevalent mutations in northeast Iran^19^. Another study showed *katG* gene mutation at codon 315 (70%) is frequently found in Iran^20^. High prevalence of mutations at codon 531 of *rpoB*^10,21–24^ and at 315 of *katG*^10,21,23,24^ have been reported from China, Vietnam, India and Nepal.

The region of gene that is 350-bp upstream of the RRDR has been reported to have mutations at codon 145, 170, 173, 174, 176, 180, 181 and 184^4,25–28^. A study conducted in China showed that the frequency of mutation at codon 531 was the highest and our study found similar results in *rpoB*^29^. The current study revealed that 67% of the treated group had mutation at codon 531 whereas 21% of the cases had mutation at codon 513 in *rpoB* gene. Therefore, the S531L substitution mutation is found to be common in RRDR. These results confirm the most prevalent mutation is found at the *rpoB* S531L and *katG* S315T codons in the Pakistani population sampled. Moreover, a novel mutation was also identified in this research where an extra Arginine was inserted at codon 513. Sequence analysis did not show any polymorphism in control strains. Studies conducted in Pakistan and China showed mutations in codon 315 of *katG*^17,18,29^ that is in agreement with our results.

Alarmingly, among 56 control group patients (unmedicated tuberculosis), 16 patients showed mutation at codon 531 in *rpoB* gene and 315 in *katG*. This fact reveals that drug resistance that was previously thought to be due to improper treatment and medication has now infected untreated patients. This striking fact may reflect the direct transfer of mutated TB strains from already infected patients. Moreover, statistical analysis showed a significant increase of MDR-TB cases among Pakistani population. So, the issue should be redressed.

It can be concluded from the results of the current study that previously reported mutations as well as novel mutations exist in the Pakistani *M. tuberculosis* strains. The current study further reveals that the most frequent mutation in *rpoB* is at the 531 codon and in *katG* at the codon 315. The double mutations in *rpoB* at positions 513 and 531 and in *katG* at 315 and 299 are notable. The presence of mutated strains in un-medicated patients is a concerning fact. Determining the mutation profile of MDR in Pakistani populations will open new horizons for the improvement of drug treatment regimens to make it more effective or for the development of novel potent drugs and vaccines to treat the drug resistance in TB. The results and molecular assessment of isoniazid and rifampicin resistance in this study were remarkable. It suggests that low-cost molecular assays may effectively be used in Pakistan for the detection of MDR-TB in clinical settings with reasonable sensitivity.

## Material and methods

### Sample collection

*M. tuberculosis* samples were collected from patients at Pakistan Medical Research Council (PMRC) Centre for Tuberculosis and Lung Diseases Mayo Hospital, Lahore. Informed consent was obtained from all subjects. Ethical Committee of Centre of Excellence in Molecular Biology, University of the Punjab approved the study. All methods were performed in accordance with the relevant guidelines and regulations.

Most specimens received by the laboratory were sputum samples. Samples were received from a total of 156 TB patients. The stringency criteria for the selection of patients was that they should have no comorbidity and should have been receiving inconsistent treatment for 2 to 5 years. 56 samples included cases of TB that had not undergone any medical treatment before and were labeled as the control group. A total of 100 samples were selected based on the long duration of the disease. These patients had received Rifampicin and Isoniazid treatment for 2–5 years inconsistently due to limited resources and poverty and were referred as the treated group. When the treatment was initiated, the treated group was not MDR. All the patients were carefully studied before and after treatment concluded. Smear slides were prepared to identify Acid Fast Bacteria to confirm the presence of *M. tuberculosis*. The selected samples were then grown on Lowenstein-Jensen (LJ) media and tested for drug susceptibility (Rifampicin and Isoniazid). The H37Rv strain (GenBank Id: AP018036.1) (a susceptible strain) was also used as a control.

### DNA extraction and PCR amplification

*M. tuberculosis* DNA was extracted from samples by following the Gentra Puregene Blood Kit (Qiagen, USA) instructions. Extracted DNA was then amplified by using the desired primers. Primer 3 was used to design the primers^30^. This study was designed to identify mutations in the two regions of *rpoB*, the RRDR and another region 350 bps upstream of the RRDR region, and in the *katG* gene of *M. tuberculosis* in Pakistani isolates. A set of primers, POB-F 5′-CGAGCTGATCCAAAACCAGA-3′ and POB-R GCTCCAGGAAGGGAATCATC, were designed to amplify a 650-bp region of *rpoB* gene and another set of primers, RRB-F 5′-CTTCTCCGGGTCGATGTCGTTG-3′ and RRB-R 5′-CGCGCTTGTCGACGTCAAACTC-3′, were designed to amplify a 365 bp region of *rpoB* gene^31^. Similarly, a region of 1120-bp of *katG* gene was amplified by Kat-F: 5′-ACTACGGGCCGCTGTTTATC-3′ and Kat-R:5′-TGAGACAGTCAATCCCGATG-3′.

The amplification conditions were the same for both POB and Kat primers: initial denaturation at 94 °C for 2 min followed by 35 cycles consisting of denaturation at 94 °C for 45 s, primer annealing at 50 °C for 45 s, primer extension at 72 °C for 3 min, followed by a final extension of 10 min. The annealing temperature used for the RRB set of primers was 64 °C for 1 min. The amplified products were visualized on a 1.5% agarose gel stained with ethidium bromide under UV transilluminator after electrophoresis.

### DNA sequencing

For DNA purification from the agarose gel, the Vivantis GF-1 Nucleic Acid Extraction Kit was used and the manufacturer’s protocol was followed. DNA samples were sequenced by using Big Dye chemistry (Applied Bioscience Inc., USA). The samples were sequenced with the related forward and reverse primers using the Applied Biosystems Prism Dye Termination method according to the manufacturer’s instructions (Big Dye Deoxy Terminators; Applied Biosystems, Weiterstadt, Germany). Sequencing was performed on an automated sequencer (Applied Biosystems; 3100 DNA Analyzer). Chromatograms of sequences were obtained and submitted to GenBank. The accession numbers of the sequences are: JN315348.1, JQ394887-JQ394890, JN626460, JN626461, JN626462, JN626463, JN626464, JN626465, JN315348, JN315349, JN315350, JN315351, JN315352, JN315353, JN315354, JN315355, JN315356, and JN626466.

### Statistical analysis

GraphPad Prism v. 8.0^32^ was used to apply Mann Whitney U test to find the probability (p-value is significant at p < 0.05) of the experiment done.

### Ethical approval

Ethical Committee of Centre of Excellence in Molecular Biology, University of the Punjab approved the study. All methods were performed in accordance with the relevant guidelines and regulations.
